# On the Validity of Evolutionary Models with Site-Specific Parameters

**DOI:** 10.1371/journal.pone.0094534

**Published:** 2014-04-10

**Authors:** Konrad Scheffler, Ben Murrell, Sergei L. Kosakovsky Pond

**Affiliations:** 1 Department of Medicine, University of California San Diego, San Diego, California, United States of America; 2 Department of Mathematical Sciences, Stellenbosch University, Stellenbosch, South Africa; Swiss Federal Institute of Technology (ETH Zurich), Switzerland

## Abstract

Evolutionary models that make use of site-specific parameters have recently been criticized on the grounds that parameter estimates obtained under such models can be unreliable and lack theoretical guarantees of convergence. We present a simulation study providing empirical evidence that a simple version of the models in question does exhibit sensible convergence behavior and that additional taxa, despite not being independent of each other, lead to improved parameter estimates. Although it would be desirable to have theoretical guarantees of this, we argue that such guarantees would not be sufficient to justify the use of these models in practice. Instead, we emphasize the importance of taking the variance of parameter estimates into account rather than blindly trusting point estimates – this is standardly done by using the models to construct statistical hypothesis tests, which are then validated empirically via simulation studies.

## Introduction

Site-to-site variation of evolutionary rates has been modeled in the phylogenetic framework using two distinct approaches, each of which has its own advantages. The first, which can be characterized as a random effects approach or hierarchical model, is based on the assumption that knowledge of the evolutionary rates at some sites can be informative for the purpose of inferring the rates at other sites. This assumption has been incorporated into models by treating site-specific rates not as free model parameters but as independent draws from a shared gene-wide distribution, which has the desirable property that data from all sites can be pooled in order to estimate it [1], [2], [3], [4], [5]. As a result, relatively complex gene-wide distributions can be estimated reliably whenever the available sequences are sufficiently long and the model is sufficiently flexible [5].

However, this strategy may be suboptimal or infeasible in some contexts and a second type of model, using what can be characterized as a fixed effects approach, has also become popular [6], [7], [8], [9]. In this approach, the rate at one site is estimated separately from the rate at another, by introducing one or more independent parameters at each site.

Empirically, fixed effects and random effects approaches yield very similar results when used in a hypothesis testing framework [6], and simulation studies [8], [9] have demonstrated expected statistical properties for hypothesis tests using models that have several site-specific parameters. For instance, the alternative models of MEDS [8] and MEME [9] for detecting sites undergoing, respectively, episodic directional and episodic diversifying selection, have four site-specific parameters each. Simulations demonstrate that tests based on these models control Type I error rates, and that their Type II error rates decrease as more sequences are included in the analysis.

Because of the limited amount of information available at a single site, it is not usually possible to obtain accurate point estimates of site-specific parameters: their confidence intervals tend to be large. This does not present a problem for statistical hypothesis tests, regardless of whether they are implemented in a Bayesian or frequentist framework, since a key feature of such tests is that the uncertainty in the parameter estimates is automatically taken into account. If the data are uninformative, the test will lack power (in a frequentist framework) or yield uninformative posteriors (in a Bayesian framework). It is therefore desirable to have some reassurance that appropriately large data sets will ensure that parameter values are estimable. Furthermore, it is desirable that hypothesis tests should be useful even for data sets of realistic size, rather than only becoming so in the limit of an infeasibly large data set.

Unfortunately, theoretical guarantees are not easily forthcoming in this context. Since the total number of site-specific parameters is proportional to the number of sites, having a large number of sites is clearly of no help when estimating the parameters of such a model. This is true even for estimates of parameters that are not site-specific: the mere presence of site-specific parameters in the model can cause estimates of *non-site-specific* parameters to be biased in the limit as the number of sites increases towards infinity – the phylogenetic analogue of the incidental parameter problem that was demonstrated in 1948 by Neyman and Scott [10]. Felsenstein [11] referred to this as the “infinitely-many-parameters” problem and pointed out that the presence of site-specific parameters can lead to unreliable inference of phylogenies. The theoretical problems underlying phylogeny inference in this context continues to receive attention [12]; here we restrict our attention to the case where the phylogeny (i.e. the tree topology and the relative branch lengths) is considered known or is inferred under a simpler model, since in practice it is typically kept fixed when site-to-site rate variation is introduced (e.g. [9]).

Intuitively, it seems reasonable that adding taxa to the data set should improve parameter estimates. However, whereas the addition of sites clearly increases the amount of information available for inference (because, in the phylogenetic models under discussion, different sites are assumed to be independent of each other), adding taxa is more complicated because the characters observed at different taxa are not independent. One can even construct pathological schemes (e.g. growing the tree by adding progressively shorter branches) by which the number of taxa can be increased without bound while the site-specific parameter estimates fail to converge. We currently have no mathematical proof even of the existence of a scheme for increasing the number of sites and taxa in such a way that site-specific parameters will converge to their true values. In a recent critique of models using site-specific parameters, Rodrigue [13] questioned the possibility of obtaining an asymptotic convergence result guaranteeing sensible behavior as data set sizes increase, claiming that one is “*left without any asymptotic conditions to envisage*”.

Given these theoretical difficulties, it is reasonable to desire empirical confirmation of the intuitive expectation that adding sequences to a data set should result in improved estimates of site-specific parameters. Here we present a simple simulation study investigating how the accuracy of the parameters estimated using these models changes as, respectively, the number of branches and sites increases.

## Methods

We simulated sequence alignments under a model with site-specific rate multipliers (Model 1) and under a model with branch-specific parameters (Model 2), investigating how the accuracy of the parameters estimated using these models changes as, respectively, the number of branches and sites increases from 64 to 128, 256, 512 and 1024. All alignments were simulated in sets of 100 replicates using balanced trees (with all branch lengths in Model 1 equal to 0.1) under the K80 model [14], transition/tranversion rate ratio  =  4.0, with site-specific rate multipliers drawn once per site (Model 1) or branch lengths drawn once per branch (Model 2) from a gamma distribution (α = β = 1) and held at the same values for all replicates. Maximum likelihood estimates were used for branch lengths in Model 2, and the site-specific rate multipliers in Model 1 were estimated using the standard approximation (e.g. [9]) of fitting relative branch lengths using all data and then holding these fixed while performing site-wise estimation of the site-specific parameters. In both cases, the tree topology was treated as known. To investigate the possibility that inferring the tree topology becomes more challenging as the number of branches increases, we also performed the Model 1 analysis using phylogenies inferred by a deliberately simplistic algorithm (neighbor joining). In the case of Model 2, it is not possible to compare true branch lengths to estimated branch lengths when the tree topologies may differ.

## Results

**Figure 1:**
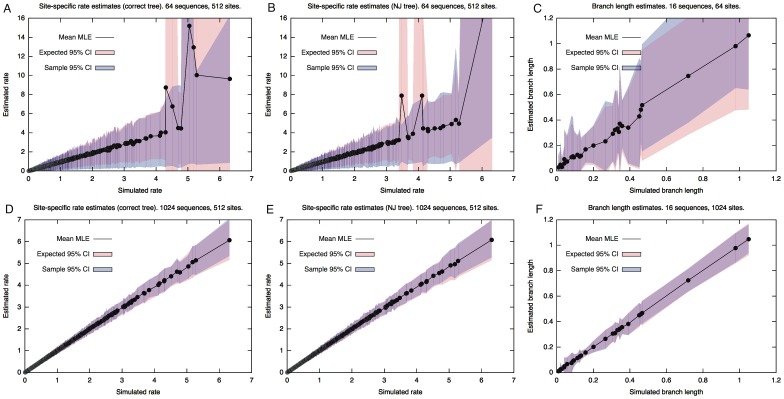
Convergence of site-specific and branch-specific parameter estimates with increasing data set size. A–B and D–E: estimated versus true parameter values for site-specific rate parameters estimated from small (A–B) and large (D–E) simulated data sets using the true tree topologies (A,D) and tree topologies inferred by the neighbor-joining algorithm (B,E). C and F: estimated versus true parameter values for branch-specific rate parameters estimated from small (C) and large (F) simulated data sets. See text for details.

Figure 1 shows the estimated versus the true parameter values for the smallest and largest data sets along with the sample confidence intervals. Also shown are the confidence intervals that are expected if the parameter estimates are normally distributed, calculated from the sample variance of the parameter estimates.

The results in Figure 1 demonstrate empirically, for one particular setting, that the estimates of site-specific parameters improve as the number of sequences increases, that this improvement occurs regardless of whether the tree topology is known a priori or inferred by neighbor-joining, and that the improvement is similar to the improvement obtained in estimated branch length parameters as the number of sites increases. In both cases, the estimates appear consistent, with variances that shrink as data set size increases. As expected, the variances of the estimates increase with the value of the rate being estimated [15]. Finally, for the largest sample size (1024 sites/sequences), the 95% confidence intervals predicted by the normal approximation to the MLE were approximately equal to the sample quantiles, suggesting that the sampling distribution of the MLE is approximately normal (as will be the case when it is close to convergence) and hence that a χ^2^ hypothesis test would be reliable.

**Figure 2:**
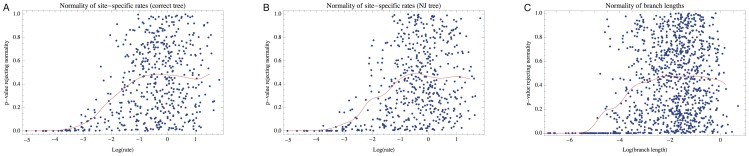
Degree of normality depends on rate and branch length. Each point on the scatter plot depicts the p-value of a Kolmogorov Smirnov test for the normality of the maximum likelihood parameter estimates. The p-value distribution corresponding to any particular rate or branch length value can be evaluated visually by considering a vertical slice through the plot. When MLEs are normally distributed, p-values will be uniformly distributed. The red curve displays the (kernel weighted) local average of the p-values, which should be near 0.5 when normality is achieved, and lower when it is rejected. For some range of true parameter values, normality is achieved for both sites (A, using the true tree topologies, and B, using tree topologies inferred by the neighbor-joining algorithm) and branches (C). However, at lower rates and shorter branch lengths, the KS test identifies systematic departures from normality, indicating that the effective sample size is likely too small for the asymptotic distribution to be reached.

When we explicitly test for normality of the MLEs using a Kolmogorov-Smirnov (KS) test (Figure 2), we see an interesting but not unexpected pattern: the ability of a normal distribution to approximate the MLEs depends on the rate (at a site) or the length (of a branch) being estimated. When the MLEs are normally distributed, the p-values from the KS test will be uniformly distributed. When MLE normality can be systematically rejected, KS p-values will be biased towards 0, and departures from uniformity are clearly visible for lower site-wise rates and for shorter branch lengths. This is reasonable because the effective sample size for estimating a rate parameter is small when divergence is low. So even though MLEs obtain normality for some site rate parameters and for some branch length parameters, suggesting they occupy the asymptotic regime, they might depart appreciably from normality for other parameters estimated on the same alignment.

## Discussion

In a recent critique of models using site-specific parameters, Rodrigue [13] presented a simulation study focusing specifically on the reliability of parameter estimates rather than on hypothesis testing. The study found that a model with site-specific parameters obtained less reliable point estimates of the parameter values than a model that describes site-to-site rate variation using gene-wide parameters, and that conclusions based on parameter point estimates obtained using the former class of models can be positively misleading. This is an important point worth emphasizing: site-specific parameters cannot be estimated reliably (at least using currently typical data sets with no more than hundreds of taxa) and models that contain such parameters are not (or should not be) developed with the aim of obtaining and interpreting point estimates of parameters. Thus, although caution is always advisable especially in the absence of theoretical guarantees, unreliable point estimates do not constitute *“inappropriate statistical properties”* as charged by Rodrigue, nor do they justify the conclusion [13] that these models “*should be approached with particular caution when the site-specific variables are high dimensional*”. Maximum likelihood point estimates have been reported as unreliable even in cases where asymptotic guarantees are available, for example in *random effects* branch site models [16], [17], and in neither class of models can the validity of hypotheses be judged solely on the basis of parameter point estimates. Instead, the models are intended for use in a statistical hypothesis testing framework (not addressed in Rodrigue's study), which takes the uncertainty in the parameter estimates into account. For instance, it is popular to use the likelihood ratio test to determine whether the hypothesis of neutrality can be rejected. In many cases one obtains an estimate of the traditional dN/dS value that is highly uncertain in the sense of having a broad confidence interval and no reliable point estimate, but for which one can nevertheless be confident that the value is larger than 1, implying positive selection. Thus unreliable point estimates cannot be interpreted as grounds to distrust hypothesis tests. We cannot blame the use of site-specific parameters when researchers choose, inappropriately, to ignore the quantifiable uncertainty in parameter estimates.

Finally, we wish to emphasize that asymptotic convergence, though desirable, is not sufficient for valid hypothesis testing in models with site-specific parameters, nor is convergence to the theoretical distribution of the test statistic necessary:

1. **Asymptotic results are not sufficient:** No asymptotic convergence result can provide us with a guarantee that inference based on a finite data set is valid. In practice, it is unclear how to establish that a given alignment is sufficiently large or informative for asymptotic results to apply or whether or not all other conditions are satisfied. The results in Figure 2 demonstrate that, even for the branch-specific parameter estimates where an asymptotic guarantee is available, the estimates obtained in a realistic scenario can converge for some branches while failing to converge for others. This is why it is standard practice to validate methods using simulation studies based on typical alignments, rather than appealing to theoretical results.
2. **Convergence is not necessary:** Likelihood ratio tests make use of the distribution of the test statistic under the null model, and for the test to be valid this distribution must be (approximately) correct. It is common to assume that conditions are appropriate for the test statistic to follow a χ^2^ distribution – if the relevant asymptotic results hold this will indeed be the correct distribution for infinitely large data sets. The success of existing methods in simulation studies also indicates that the χ^2^ distribution is a good approximation when those methods are applied to alignments of typical size (i.e. sizes similar to those used in the simulations). If, however, for a model with site-specific parameters, the χ^2^ distribution is found to be a poor approximation, this would not invalidate the use of such models but merely imply that a better approximation (perhaps an estimate obtained via the parametric bootstrap [9]) is required.

In conclusion, in the absence of theoretical results, our simulation results provide empirical reassurance that additional taxa do provide additional information that is accessible to models with site-specific parameters, and that such models can produce useful parameter estimates for realistically sized data sets. We wish to emphasize the importance of empirically validating hypothesis tests based on these models, but see no reason to distrust them once such validation has been performed.
